# Toward an information theoretical description of communication in brain networks

**DOI:** 10.1162/netn_a_00185

**Published:** 2021-08-30

**Authors:** Enrico Amico, Kausar Abbas, Duy Anh Duong-Tran, Uttara Tipnis, Meenusree Rajapandian, Evgeny Chumin, Mario Ventresca, Jaroslaw Harezlak, Joaquín Goñi

**Affiliations:** Institute of Bioengineering, Center for Neuroprosthetics, EPFL, Geneva, Switzerland; Department of Radiology and Medical Informatics, University of Geneva (UNIGE), Geneva, Switzerland; Purdue Institute for Integrative Neuroscience, Purdue University, West Lafayette, IN, USA; School of Industrial Engineering, Purdue University, West Lafayette, IN, USA; School of Industrial Engineering, Purdue University, West Lafayette, IN, USA; Purdue Institute for Integrative Neuroscience, Purdue University, West Lafayette, IN, USA; School of Industrial Engineering, Purdue University, West Lafayette, IN, USA; School of Industrial Engineering, Purdue University, West Lafayette, IN, USA; Psychological and Brain Sciences, Indiana University, Bloomington, IN, USA; School of Industrial Engineering, Purdue University, West Lafayette, IN, USA; Department of Epidemiology and Biostatistics, Indiana University, Bloomington, IN, USA; Purdue Institute for Integrative Neuroscience, Purdue University, West Lafayette, IN, USA; School of Industrial Engineering, Purdue University, West Lafayette, IN, USA; Weldon School of Biomedical Engineering, Purdue University, West Lafayette, IN, USA

**Keywords:** Human connectome, Communication regimes, Brain connectomics, Information theory, Brain networks

## Abstract

Modeling communication dynamics in the brain is a key challenge in network neuroscience. We present here a framework that combines two measurements for any system where different communication processes are taking place on top of a fixed structural topology: path processing score (PPS) estimates how much the brain signal has changed or has been transformed between any two brain regions (source and target); path broadcasting strength (PBS) estimates the propagation of the signal through edges adjacent to the path being assessed. We use PPS and PBS to explore communication dynamics in large-scale brain networks. We show that brain communication dynamics can be divided into three main “communication regimes” of information transfer: *absent communication* (no communication happening); *relay communication* (information is being transferred almost intact); and *transducted communication* (the information is being transformed). We use PBS to categorize brain regions based on the way they broadcast information. Subcortical regions are mainly direct broadcasters to multiple receivers; Temporal and frontal nodes mainly operate as broadcast relay brain stations; visual and somatomotor cortices act as multichannel transducted broadcasters. This work paves the way toward the field of brain network information theory by providing a principled methodology to explore communication dynamics in large-scale brain networks.

## INTRODUCTION

Deciphering communication dynamics in the human brain is one of the biggest open challenges in modern neuroscience (Avena-Koenigsberger et al., 2018). Communication in the brain can be measured and modeled at different spatial scales: starting from the fine-grained microscale exploration of information transfer between neuronal spikes (Quian Quiroga & Panzeri, 2009; Timme & Lapish, 2018), to inferring communication at mesoscale from electrical activity of cortical populations (Laughlin & Sejnowski, 2003; Nigam et al., 2016), up to macroscale brain networks estimated from in vivo magnetic resonance imaging (MRI) data; the latter being the focus of this work.

Particularly, in large-scale (MRI-based) brain networks, many hurdles have made the investigation of brain communication challenging. One issue arises from data acquisition, which outputs noisy and indirect measurements of neuronal activity (and subsequent connectivity or information transfer). Another issue is the difficulty of validating in silico brain communication models, although meaningful progress has been made (see Aerts et al., 2018; Cabral et al., 2017; Glomb et al., 2017; Ritter et al., 2013; Sanz Leon et al., 2013). Additionally, several methodological factors such as selection of temporal scales, frequency ranges, time windows, and time-varying or lagged dependencies can have significant impact on assessment of brain communication dynamics (Avena-Koenigsberger et al., 2018).

Nonetheless, in the last two decades, improvements in MRI hardware and development of new data acquisition sequences have allowed for application of methodologies from graph theory and dynamical systems, giving rise to the field of network neuroscience or brain connectomics (Bassett & Sporns, 2017; Fornito et al., 2016). In brain connectomics, the investigation of functional and structural connections in the human brain is modeled using tools and methods from network science (Fornito et al., 2016; Sporns, 2010). Structural connections between brain region pairs are modeled from diffusion-weighted imaging data, denominated as structural connectome or structural connectivity (SC). Functional connections are modeled from functional magnetic resonance imaging data (fMRI), by measuring temporal statistical dependences between brain region pairs, usually defined as functional connectivity or functional connectome (FC). Examining human brain connectivity data offers new insights on how the integration and segregation of information in the brain relates to human behavior (Deco et al., 2015; Sporns, 2013), and how network organization may be altered in neurological diseases and disorders (Bassett & Bullmore, 2009; Fornito et al., 2015; Rosazza & Minati, 2011; Stam, 2014).

Brain connectomics has provided a proper mathematical framework upon which network neuroscientists have begun to layout several alternative models to capture and explain the complex patterns of brain communication dynamics stemming from large-scale brain networks. Pioneering work started by assessing the link between network topology and communication, from routing-based models with full knowledge of the topology of the brain network (i.e., signaling along shortest paths, de Pasquale et al., 2016; Graham, 2014), to diffusion models “uninformed” of the topology of the network (Abdelnour et al., 2014; Raj et al., 2012). Hybrid models exploring a spectrum of communication dynamics, including search information (Goñi et al., 2013, 2014), navigation (Seguin et al., 2018), or k-shortest path ensembles (Avena-Koenigsberger et al., 2017), have also been investigated. Recent studies have also looked into alternative network communication measures such as Markovian queuing networks (Mišić et al., 2014b), linear transmission models of spreading dynamics (Mišić et al., 2015; Worrell et al., 2017), cooperative learning (Tipnis et al., 2018), and diffusion processes based on memory-biased random walks (Masuda et al., 2017), as well as studying asymmetries of communication in large-scale brain networks (Seguin et al., 2019).

Despite all these efforts in the development of communication models that explain human brain dynamics (Hahn et al., 2019; Joglekar et al., 2018), there is a lack of a principled theory of brain network communication, which aims to address the following question: how can one characterize the multifold communication regimes originating in the brain, on top of a fixed physical constrain represented by its structural connections?

As a matter of fact, human brain connectivity can be modeled by a multilayered complex network that contains one slowly evolving structural topology (its structural connectome) and one rapidly evolving task-dependent functional architecture (its FC) (Amico et al., 2019; Cole et al., 2014). In this context, there is a lack of a well-grounded mathematical framework that can associate structural and functional patterns and quantify the many facets of communication dynamics.

Here, we introduce a framework that combines two information-theoretical measurements for any system where different communication processes are taking place over a fixed structural topology. The first measurement, path processing score (PPS), estimates how much the brain signal has changed or transformed on a path between a source and a target brain region. A negative score is indicative of a path that is not being used for communication, a PPS around zero indicates that information is passed almost intact along a path from the source to the target, whereas a high PPS indicates that the signal has gone through considerable transformation. The second measurement, path broadcasting strength (PBS), estimates the propagation of the signal through the edges adjacent to the path being assessed. A low PBS indicates a *routing-based* communication along a path, whereas a high PBS indicates that the communication is not specific to that path, but is also being *broadcast* or propagated through neighboring edges.

We apply these two measurements to investigate the communication dynamics in resting-state and task functional MRI (fMRI) of 100 unrelated subjects from the Human Connectome Project (HCP). By assessing PPS, we show that routing communication dynamics in large-scale brain networks can be separated into three main “regimes”: *absent communication,* where no communication is happening along that path; *relay communication*, where communication is specific to that path (i.e., unchanged or minimally changed brain signal); and *transducted communication*, where communication is not path specific (i.e., transformed; modified brain signal). In addition to these three regimes, we show that our second metric, PBS, can quantify the spread of information transfer around the path (i.e., routing or diffused communication/broadcasting).

The information theoretical framework presented here allows for the joint assessment of structural and functional connectivity and has revealed different communication regimes across brain regions and different cognitive tasks. Furthermore, it also revealed a regional specificity in the way the brain broadcasts information, by categorizing brain regions into three main “communication modalities”: *direct broadcasters* to multiple receivers (predominantly subcortical regions); *broadcast relay brain stations* (mainly limbic system); and, finally, *multichannel transducted broadcasters* (mainly visual and somatomotor cortices).

This investigation was motivated by a need to better understand communication dynamics in large-scale brain networks, and it was partly inspired by the seminal masterpiece by Claude Shannon (Shannon, 1948). Several studies have shown functional connectivity changes across fMRI conditions (Amico et al., 2019, 2020; Cole et al., 2014; Gonzalez-Castillo & Bandettini, 2018; Mohr et al., 2016; Schultz & Cole, 2016). In other words, there is an adaptation or functional reconfiguration that occurs as subjects perform different tasks and/or switch between different cognitive modes (Gilson et al., 2018; Gonzalez-Castillo & Bandettini, 2018; Shine et al., 2016; Shine & Poldrack, 2018). In this paper, we further investigate how those changes can be reflected by communication regimes tracked on underlaying SC paths. With this work, we introduce a new framework based on information theoretical principles to infer the basic units of information transfer in large-scale human brain networks, as well as to assess how they change and evolve between subjects or across cognitive tasks.

## METHODS

### Dataset

The dataset of functional and structural neuroimaging data used in this work came from the Human Connectome Project (HCP, https://www.humanconnectome.org/), Release Q3. Per HCP protocol, all subjects gave written informed consent to the HCP consortium. These data contained fMRI and diffusion-weighted imaging (DWI) acquisitions from 100 unrelated subjects of the HCP 900 data release (Van Essen et al., 2012, 2013). All HCP scanning protocols were approved by the local Institutional Review Board at Washington University in St. Louis.

### HCP: fMRI Acquisition

We used fMRI runs from the 100 unrelated subjects of the HCP 900 subjects data release (Van Essen et al., 2012, 2013). The fMRI resting-state runs (HCP filenames: rfMRI_REST1 and rfMRI_REST2) were acquired in separate sessions on two different days, with two different acquisitions (left to right or LR and right to left or RL) per day (Glasser et al., 2013; Van Essen et al., 2012, 2013). The seven fMRI tasks were the following: gambling (tfMRI_GAMBLING), relational (tfMRI_RELATIONAL), social (tfMRI_SOCIAL), working memory (tfMRI_WM), motor (tfMRI_MOTOR), language (tfMRI_LANGUAGE, including both a story-listening and arithmetic task), and emotion (tfMRI_EMOTION). The working memory, gambling, and motor tasks were acquired on the first day; all other tasks were acquired on the second day (Barch et al., 2013; Glasser et al., 2013). For all sessions, data from both the LR and RL phase-encoding runs were used to calculate connectivity matrices and averaged together. Full details on the HCP dataset have been published previously (Barch et al., 2013; Glasser et al., 2013; S. M. Smith et al., 2013).

### HCP: DWI Acquisition

We used DWI data from the same 100 unrelated subjects of the HCP 900 subjects data release (Van Essen et al., 2012, 2013). The diffusion-weighted (DW) acquisition protocol is covered in detail elsewhere (Glasser et al., 2013; Sotiropoulos et al., 2013). Below we mention the main characteristics. Very high resolution acquisitions (1.25 mm isotropic) were obtained by using a Stejskal–Tanner (monopolar) (Stejskal & Tanner, 1965) diffusion-encoding scheme. Sampling in q-space was performed by including three shells at b = 1,000, 2,000 and 3,000 s/mm^2^. For each shell, a corresponding 90 diffusion gradient directions and 5 b0 volumes were acquired twice, with the phase-encoding (PE) direction reversed for each pair (i.e., LR and RL pairs). Directions were optimized within and across shells (i.e., staggered) to maximize angular coverage using the approach of Caruyer et al. (2011) (https://www-sop.inria.fr/members/Emmanuel.Caruyer/q-space-sampling.php) and form a total of 270 noncollinear directions for each PE direction. Correction for echo planar acquisition and eddy-current-induced distortions in the diffusion data was based on manipulation of the acquisitions so that a given distortion manifests itself differently in different images (Andersson et al., 2003). To ensure better correspondence between the PE reversed pairs, the whole set of DW volumes was acquired in six separate series. These series were grouped into three pairs, and within each pair the two series contained the same DW directions but with reversed phase encoding (i.e., a series of DW volumes with RL phase encoding is followed by a series of volumes with LR phase encoding).

### Brain Parcellation

We employed a cortical parcellation of 360 brain regions, as recently proposed by Glasser et al. (2016) for definition of brain network nodes. For completeness, 14 subcortical regions were added, as provided by the HCP release (filename “Atlas_ROI2.nii.gz”), as analogously done in previous papers (Amico et al., 2019; Amico & Goñi, 2018a, 2018b). To do so, this file was converted from NIFTI to CIFTI format by using the HCP workbench software (Glasser et al., 2013; Marcus et al., 2011) (command *-cifti-create-label* https://www.humanconnectome.org/software/connectome-workbench.html).

### HCP: fMRI Preprocessing

Data were processed following the HCP functional preprocessing guidelines (Glasser et al., 2013; S. M. Smith et al., 2013). Briefly, processing steps included artifact removal, motion correction, and registration to standard Montreal Neurological Institute space in both volumetric and grayordinate formats (i.e., where brain locations are stored as surface vertices; S. M. Smith et al., 2013), with weak highpass temporal filtering (>2,000s full width at half maximum) applied to both formats, for slow drift removal. MELODIC ICA (Jenkinson et al., 2012) was applied to volumetric data, and artifact components were subsequently identified using FSL-FIX (Salimi-Khorshidi et al., 2014). Artifacts and motion-related time courses (i.e., the six rigid-body parameter time series, their backward-looking temporal derivatives, plus all 12 resulting regressors squared) were then regressed out of both volumetric and grayordinate data (S. M. Smith et al., 2013).

For the resting-state fMRI data, we also added the following steps (Amico et al., 2019; Amico & Goñi, 2018a, 2018b): global gray matter signal was regressed out of the voxel time courses (Power et al., 2014); a bandpass first-order Butterworth filter in forward and reverse directions [0.001 Hz, 0.08 Hz] was applied (Matlab functions *butter* and *filtfilt*); voxel time courses were *z*-scored and then averaged per brain region, excluding outlier time points outside of 3 standard deviation from the mean, using the workbench software (Marcus et al., 2011) (workbench command *-cifti-parcellate*). For task fMRI data, we applied the same steps, with exception of a less restrictive range for the bandpass filter [0.001 Hz, 0.25 Hz].

Functional connectivity network edge weights were defined as mutual information (Cover & Thomas, 2012; Shannon, 1948) between all node pairs, calculated by uniform binning of the *z*-scored BOLD time courses (bin widths = 0.5 standard deviation, spanning range = [−3.5 3.5] *z*-scored BOLD activation). This resulted in a positive symmetric connectivity matrix for each fMRI session of each subject. On top of using MI-bin equal to 0.5, we have also explored three additional *z*-score bin widths (0.75, 1, and 2) within the *z*-score range [−3.5 to 3.5]. This binning procedure was applied to the *z*-scored BOLD time series before computing mutual information. Results shown in the Supporting Information Table S1 indicate that MI pairwise measurements are stable across different bin sizes. Functional connectivity matrices from the LR and RL phase-encoding runs were averaged to improve signal-to-noise ratio (as done in Finn et al., 2015). The functional connectomes were kept in its weighted form (as measured by mutual information), hence neither thresholded nor binarized.

Finally, the resulting individual functional connectivity matrices were ordered (rows and columns) according to seven resting-state cortical networks, as proposed by Yeo et al. (2011). For completeness, an eighth subnetwork including the 14 HCP subcortical regions was added (as analogously done in recent papers: Amico et al., 2019; Amico & Goñi, 2018a, 2018b).

### HCP: DWI Preprocessing

The HCP DWI data were processed following the MRtrix3 (Tournier, Calamante, & Connelly, 2012) guidelines (for the full documentation see https://mrtrix.readthedocs.io/en/latest/tutorials/hcp_connectome.html). The following were carried out: (a) generation of a tissue-type segmented image appropriate for anatomically constrained tractography (MRtrix command *5ttgen*; R. E. Smith et al., 2012); (b) estimation of the multishell multitissue response function (MRtrix command *dwi2response msmt_5tt*; Christiaens et al., 2015); (c) multishell, multitissue constrained spherical deconvolution (MRtrix *dwi2fod msmt_csd*; Jeurissen et al., 2014); (d) generation of the initial tractogram (MRtrix command *tckgen*, 10 million streamlines, maximum tract length = 250, FA cutoff = 0.06); and (e) application of the second version of Spherical-deconvolution Informed Filtering of Tractograms (SIFT2; R. E. Smith et al., 2015) methodology (MRtrix command tcksift2). Both SIFT (R. E. Smith et al., 2013) and SIFT2 (R. E. Smith et al., 2015, p. 2) methods provide more biologically meaningful estimates of structural connection density. However, SIFT2 allows for a more logically direct and computationally efficient solution to the streamlines connectivity quantification problem: by determining an appropriate cross-sectional area multiplier for each streamline rather than removing streamlines altogether, biologically accurate measures of white matter fiber connectivity are obtained while making use of the complete streamlines reconstruction (R. E. Smith et al., 2015). SIFT2 obtained streamlines were then mapped onto the 374 chosen brain regions (see Brain Parcellation Atlas section for details), and the average streamline length (millimeters) was calculated for all brain regions pairs (MRtrix command *tck2connectome*). Henceforth, what we will refer to as “structural connectome” represents the physical distance (in millimeters) between brain regions pairs. Here we opted for the streamline length in this case, because we wanted to link information transfer in brain networks with the sender-channel-receiver schematics proposed in electronic communication by Shannon (1948). Therefore, we approximated the concept of communication channels to shortest paths based on structural fiber length in brain networks (Bullmore & Sporns, 2012).

### Mathematical Foundations of Communication in Large-Scale Brain Structural-Functional Networks

There are two main fundamental assumptions behind the framework we are proposing here. First, in order to transfer (send or receive) information directly, two brain nodes must be *structurally connected* through white matter fibers (or streamlines, as obtained through tractography); second, the amount of communication taking place between two structurally connected nodes can be estimated as the functional coupling between them, here measured as the *mutual information* (Cover & Thomas, 2012) between the corresponding BOLD time series.

In summary, we define two brain regions as “communicating” when they are structurally connected and their correspondent time series show statistical dependence, with the amount of “communication” being measured through pairwise mutual information. Please note that we are using the word communication here in Shannon’s sense, that is, we are trying to characterize the amount of information shared between sent and received fMRI BOLD signals, as measured through mutual information.

Starting from these two assumptions, we here lay the basis for an information-theoretical evaluation of communication following (structural) *shortest paths* in human large-scale brain networks. Note, however, that although this work focused on communication along shortest paths, the proposed framework can be generalized to any existing path.

### Assessment of Well-Behaved Communication Along Shortest Paths

Part of the conceptualization of this framework was strongly inspired by seminal work by Claude Shannon, “*A Mathematical Theory of Communication*,” particularly one main concept stemming from that work: the concept of data processing inequality (DPI; Cover & Thomas, 2012). In brief, the DPI theorem states that in a Markov chain of three random variables X, Y, Z, where X→Y→Z, then MI(X;Y) ≥ MI(X; Z), where MI(X; Y) and MI(X; Z) denote the mutual information between X and Y and between X and Z, respectively. Note that this theorem can be easily extended to chains larger than N = 3 (Cover & Thomas, 2012).

In other words, processing Y cannot add new information about X. This theorem has a reasonable analogy. Think of the children’s “telephone game”. Briefly, players form a line, and the first player comes up with a message and whispers it to the second player in line. The second player repeats the message to the third player, and so on. In those conditions, the message sent to player Z through “middle player” Y can never be more intact than the original version sent by the first player X, at most equal or worse (i.e., player Y might mishear player X and alter the message).

Inspired by the concept of DPI on a chain, we defined a novel brain network measure, the PPS. Let $\Pi_{s\to t}^{\text{task}}$ be the shortest path between a brain region source (S) and a brain region target (T) for a specific fMRI *task* (e.g., resting state, language, etc.). We defined such shortest path as a sequence of nodes Ω_*s*→*t*_ = {*S*, *K*_1_, *K*_2_, …, *K*_*m*_, *T*}, starting at the source S and ending at the target T, with *m* intermediate nodes in between. Let us define also Ω_s*→*t*_ = {*K*_1_, *K*_2_, …, *K*_*m*_, *T*} and Ω_*s*→t*_ = {*S*, *K*_1_, *K*_2_, …, *K*_*m*_} as the sequences of shortest path nodes without the source and the target on the structural connectome (SC), respectively. The SC reference model used in this study is based on fiber length. Please note that such SC was not binarized or thresholded. The shortest path computation is entirely based on the group-averaged positive-weighted matrix defined by pairwise fiber lengths.

Note also that $\Pi_{s\to t}^{\text{task}}$ is a *structural shortest path* (i.e., obtained from the structural connectome), where weights {*S* → *K*_1_ → *K*_2_ → … → *K*_*m*_ → *T*} along the path are substituted by the *mutual information values* calculated on the FC, that is, {*MI*(*S*; *K*_1_), *MI*(*K*_1_; *K*_2_) … *MI*(*K*_*m*−1_; *K*_*m*_), *MI*(*K*_*m*_; *T*)}. Each term represents the mutual information between the fMRI time series of brain regions along the structurally connected shortest path for a specific task.

The PPS of a structural shortest path associated with a specific functional task is then defined as:

$$PPS\left(\Pi_{s\to t}^{\text{task}}\right)=\sum_{i\in \Omega_{s*\to t}}\left(MI\left(S;K_{1}\right)-MI\left(S;i\right)\right)$$

In a nutshell, PPS estimates how much the signal has changed or been transformed between any source and target in the brain network. In a sense, it is a relaxation of the DPI, a more qualitative measurement than the Shannon’s “strict” data processing theorem. This choice is based on the idea that in human MRI brain networks, it is extremely likely that communication between two brain regions can happen on nonshortest paths (Avena-Koenigsberger et al., 2017; Goñi et al., 2014; Tipnis et al., 2018). Therefore, a score such as PPS allows for a more flexible exploration of the communication dynamics underlying the fixed structural topology. Note that PPS is not defined for pairs of brain regions with a shortest path that consists of one edge. Also note that PPS is a nonsymmetric measurement, that is, PPS(s→t) ≠ PPS(t→s).

As a simple example, let us assume a shortest path defined as a simple chain of three nodes (source S, intermediate node X, target T). PPS in this case is simply defined as: *PPS*(Π_*s*→*t*_) = *MI*(*S*; *X*) − *MI*(*S*; *T*). If this difference is positive, it means that the mutual information along the path has decreased, which then can be understood as an attenuation of the signal (or increase in noise). However, if the difference is negative, that would mean that the DPI is not satisfied. In that case, it adds evidence that the communication between S and T might not be traveling along that path (e.g., that communication path is unlikely to be in use for regions S and T).

**Figure 1.**
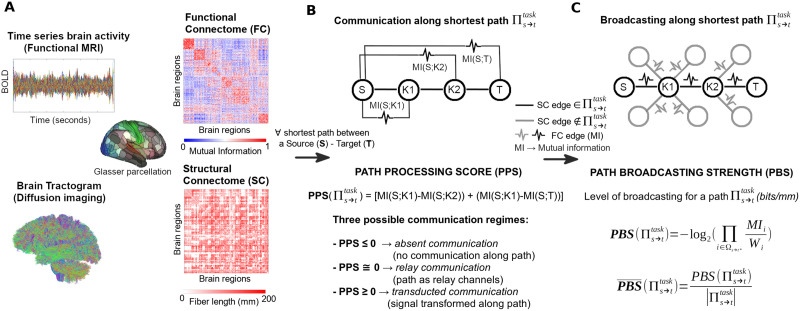
Toward a mathematical theory of communication for the human connectome. (A) Functional and structural connectomes are extracted from brain data for a multimodal brain parcellation (Glasser et al., 2016). (B) For every shortest path between a source-target pair of brain regions, path processing score (PPS) is computed and the path is assigned to its correspondent communication regime. (C) For each communication regime, path broadcasting strength (PBS) is evaluated to determine the spread of information across the shortest path.

The evaluation of PPS for a shortest path can tell us a lot about the communication regime taking place between source region S and target region T (see Figure 1B). For instance, a low (or close to zero) PPS indicates that information is passed almost intact from the source to the target: hence, we are in presence of a relay communication regime. Conversely, a high-processing load indicates that the signal has gone through considerable transformations (due to either internal or external inputs): the shortest path is then operating in a transducted communication regime. Finally, if the PPS is negative, it means that, despite the relaxation of the DPI theorem, communication along the shortest path is absent, that is, the mutual information along the path increases with respect to the mutual information of the original message.

### Assessment of Information Broadcasting Along Shortest Paths

Search information (SI) quantifies the *hiddenness* of the shortest path between a source node and a target node within the network by measuring the amount of knowledge or information in bits needed to access the path (Goñi et al., 2014; Rosvall et al., 2005; Sneppen et al., 2005). The more nested the shortest path between two brain regions, the higher its SI value. Conversely, the less hidden or integrated the path, the lower its SI value.

Inspired by this concept, we defined a measure of PBS. Similarly to the PPS defined earlier, PBS is measured as the SI (Goñi et al., 2014) along the structural shortest path $\Pi_{s\to t}^{\text{task}}$, but superimposing the functional weights corresponding to pairwise mutual information between the brain regions along the structural path. Hence, let *MI*_*s*→*t*_ = {*MI*(*S*; *K*_1_), *MI*(*K*_1_; *K*_2_), …, *MI*(*K*_*m*−1_; *K*_*m*_), *MI*(*K*_*m*_; *T*)} be the set of mutual information values along the shortest path and *W* = {*w*_*S*_, *w*_*K*1_, *w*_*K*2_ … *w*_*Km*_, *w*_*T*_} be the set of the nodal strength along the shortest path (again, note that the nodal strength is calculated from the mutual information values where a structural edge is present). Nodal strength of a brain region i is defined as *W*_*i*_ = Σ_*j*_ MI_*ij*_, for all *j* ≠ *i*, which sums all functional connectivity values in which brain region *i* participates.

We can then define the PBS as:

$$PBS\left(\Pi_{s\to t}^{\text{task}}\right)=-\log_{2}\left(\prod_{i\in \Omega_{s\to t*}}\frac{{MI}_{s\to t}^{i}}{W_{i}}\right)$$

where *W*_*i*_ refers to the *i*-th element of the ordered sequence of nodal strengths along the path $\Pi_{s\to t}^{\text{task}}$. Analogously, ${MI}_{s\to t}^{i}$ refers to the *i*-th element of the *MI*_*s*→*t*_ ordered sequence along the path $\Pi_{s\to t}^{\text{task}}$. This equation does not take into account the bias arising from different path lengths. That is, longer shortest paths will have a tendency to yield higher PBS values. To account for this, we therefore normalize PBS:

$$\bar{PBS}\left(\Pi_{s\to t}^{\text{task}}\right)=\frac{PBS\left(\Pi_{s\to t}^{\text{task}}\right)}{\left|\Pi_{s\to t}^{\text{task}}\right|}$$

where |$\Pi_{s\to t}^{\text{task}}$| is the total sum of the shortest path length (in millimeters, in this case). Henceforth, what we will refer to as PBS is its normalized version. PBS is essentially the SI (Goñi et al., 2014) computed on the functional values superimposed on a fixed structural topology (Figure 1C). However, conceptually the interpretation differs. In fact, measuring SI on functional edges allow us to investigate how communication propagates along shortest paths. For instance, when PBS is low, the signal is flowing primarily along the shortest path, hence communication between source and target regions takes place through a *routing mode*. Conversely, when PBS is high, the communication between a regions pair is being propagated through edges adjacent to the shortest path as well, hence operating in a *broadcasting mode*.

Therefore, we can associate to each of the two *communication regimes* defined through PPS (i.e., *relay* and *transducted*), as well as for (structurally) directly connected nodes (i.e., *direct communication*), its corresponding *communication mode* (*routing* or *broadcasting*), for any shortest path between a brain region source S and a target T (Table 1; see also Figure 1). Note that, by defining edge weights as mean streamline length (in millimeters), the resultant units of PBS are bits/mm.

**Table 1.** Schematic of the different *communication regimes* based on the path processing score (PPS) measurement, and their associations to the spread of information (*communication mode*) along the shortest path, as assessed through path broadcasting strength (PBS)

**Communication regime (PPS)**	**Broadcasting level (PBS)**	**Communication mode**
**Direct communication (single-edge shortest path, PPS not defined)**	***Low broadcasting*** → *Single-edge routing*	
	***High broadcasting*** → *Multi-edge routing*	
**Absent communication (PPS < 0)**	*No broadcasting No communication along shortest path along shortest path*	
**Relay communication (PPS ≅ 0)**	***Low broadcasting** → Routing relay path*	
	***High broadcasting** → Broadcasting relay path*	
**Transducted communication (PPS > 0)**	***Low broadcasting** → Routing transduction*	
	***High broadcasting** → Broadcasting transduction*	

Note that PBS is a 374 × 374 nonsymmetric matrix, since every source-target pair in the brain network has a PBS score. Hence, based on PBS, we define two different nodal broadcasting strengths, differentiating when a brain region *k* is a *sender* (*WBS*_*sender*_(*k*)) or a *receiver* (*WBS*_*receiver*_(*k*)):

$${WBS}_{sender}\left(k\right)=\sum_{i}^{N}{PBS}_{ik};\;{WBS}_{receiver}\left(k\right)=\sum_{i}^{N}{PBS}_{ki}$$

where *N* = 374 (number of brain regions). Finally, we define the (symmetric) nodal broadcasting strength (WBS) as the average, per brain region *k*, of both measurements:

$$WBS\left(k\right)=\frac{{WBS}_{\text{sender}}\left(k\right)+{WBS}_{\text{receiver}}\left(k\right)}{2}$$

### DMN-Based Model for Identification of Communication Regimes

We defined the boundaries of the relay communication regime based on the PPS distribution obtained by considering only all pairwise within default mode network (DMN) interactions. The DMN at rest is a highly coherent integrated functional network. Hence we used DMN at rest for setting the boundaries of relay communication with respect to broken and transducted communication. Therefore, for each subject, we obtained the DMN-based shortest paths and their corresponding PPS for resting state. Finally, the boundaries for a PPS to be considered “close to zero” or in *relay communication* were set to the [5, 95] percentiles of the DMN-based distribution, specifically to the PPS range [−0.04 0.07] (see Supporting Information Figure S1).

Here we used PPS and PBS to investigate, respectively, the communication regimes and communication modes of large-scale brain networks in 100 HCP subjects, for resting state and seven different cognitive tasks (see HCP: fMRI Acquisition section for details). The scheme depicted in Figure 1 provides a summary of these two information-theoretical measurements of brain communication.

## RESULTS

**Figure 2.**
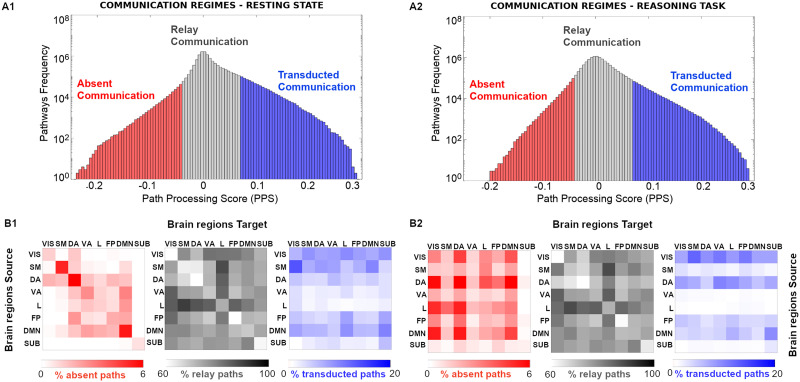
Communication regimes in large-scale brain networks. (A1 and A2) Path processing score (PPS) on indirect pathways allows to separate brain network communication in three different regimes: absent, relay communication, and transducted communication). Note that a common SC reference (group average) was used to estimate shortest paths based on fiber length (see more details in Methods section). (B1 and B2) The percentage of paths, for the three different communication regimes, corresponding to the within and between seven functional networks source-target pairs, as specified by Yeo et al. (2011). An eighth subcortical set was added for completeness.

We evaluated communication dynamics in large-scale human brain networks obtained from the MRI dataset of 100 unrelated subjects (Van Essen et al., 2013) under resting-state and task conditions. Task results presented in Figure 2A2 and 2B2 refer to the reasoning task (see Supporting Information Figure S2 for the other 6 fMRI tasks). First, we used the PPS measurement (see Methods section) to characterize the shortest paths based on the three different communication regimes (*absent*, *relay*, and *transducted*; Figure 2A1 and A2). Note that the boundaries for the communication regimes were calculated from a DMN-based PPS distribution obtained from resting-state data (for details see Methods: DMN-Based Model section for communication regimes; Supporting Information Figure S1 shows the distribution obtained with dashed vertical lines indicating 5 and 95 percentiles). Also note that even though we decided in this work to focus on the group-averaged connectomes (and trials), PBS and PPS are very stable when compared across subjects and source-target pair (see Supporting Information Table S2).

For each of the three different communication regimes, we stratified shortest paths into the seven functional networks as defined by Yeo et al. (2011) (adding the subcortical set as in Amico et al., 2017b) to investigate whether communication regimes were functional networks specific. We observed interesting structure in the distribution of communication pathways per functional network (Figure 2): the limbic system seems to be a hub for the relay communication regime for both task and resting-state connectomes, while the streams toward visual and DMN modules are mostly present at the transducted communication regime for both resting-state and reasoning task (Figure 2B1 and B2, gray and blue patterns; the same applies to the other tasks, see Supporting Information Figure S2). Notably, for absent paths, differential patterns emerged for the resting-state and reasoning task, where absent paths predominantly appeared within network at rest and between networks during the reasoning task (Figure 2B1 and B2, red patterns). A similar pattern was observed for the other task conditions (Supporting Information Figure S2). This might be related to the tendency of going out of the optimal “routing” strategy (preferential in resting) when switching to a cognitive task.

**Figure 3.**
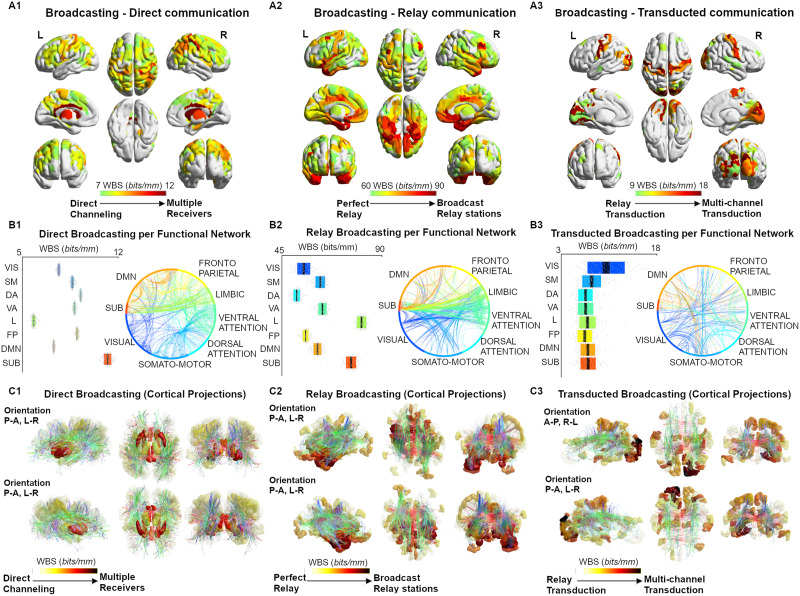
Broadcasting in large-scale brain networks during rest. (A1–A3) Nodal broadcasting strength (WBS, measured in bits/mm; see Methods section for details) shown for the top 100 brain regions for the three different communication regimes (direct communication, relay communication, transducted communication). (B1–B3) Broadcasting properties evaluated for each of the seven functional networks specified by Yeo et al. (2011). An eighth subcortical community was added for completeness. (C1–C3) The broadcasting matrices are projected onto brain renders, where tracts (color coded by direction; red: left-right; green: anterior-posterior; blue: superior-inferior) represent nonzero edges in the masks, and nodal strength (A1–A3) is mapped onto the cortical meshes from low WBS (white, transparent) to high WBS (opaque, bright red).

We further characterized shortest path communication regimes into two *communication modes* (routing or broadcasting) based on our second proposed metric, the PBS (see Methods and Table 1 for details). PBS quantifies the degree to which information would propagate solely along the shortest path (routing) or spread out to nodes branching from the shortest path (broadcasting). In addition to the relay and transducted regimes, which constitute paths of at least two hops, PBS was also evaluated on direct (one hop) paths (here referred to as the *Direct Communication* regime, Table 1). Notably, regional specificity emerged at each level of broadcasting (computed as nodal broadcasting strength or WBS; see Methods) (Figure 3A1–3A3). Specifically, within the direct communication regime, paths that involved subcortical nodes (as source/target) displayed the highest degree of broadcasting (Figure 3A1, 3B1, and 3C1; average nodal PBS of 12 bits/mm). For the relay communication regime, paths from/to the limbic and subcortical regions had the highest PBS (∼90 bits/mm), operating as broadcast relay stations (Figure 3A2, 3B2, and 3C2) while, in the transducted regime pathways, visual and somatomotor cortices were the hubs of broadcasting transduction (Figure 3A3, 3B3, 3C3; PBS ∼ 15 bits/mm).

**Figure 4.**
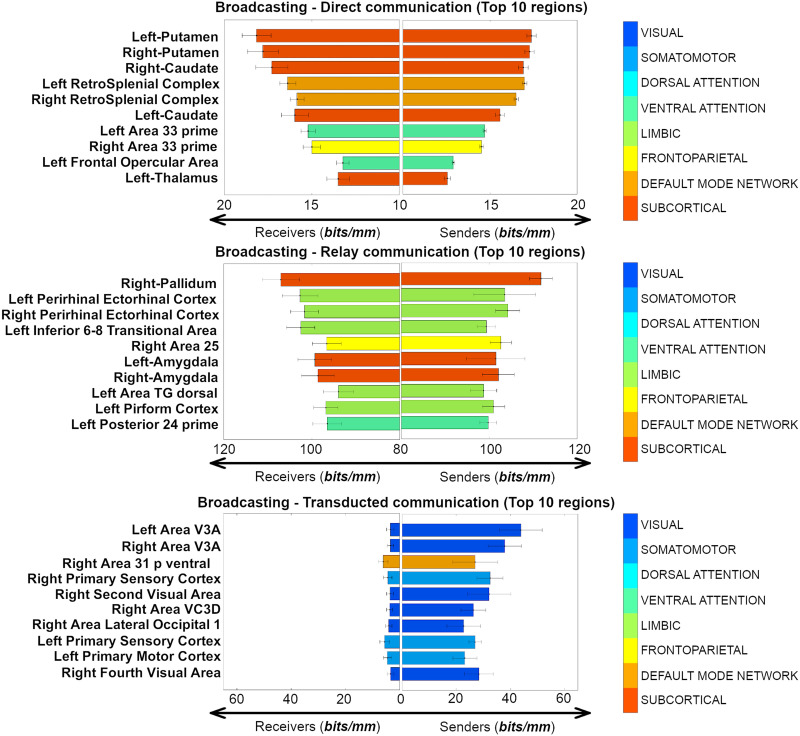
Anatomical sender-receiver list of the top 10 brain regions involved in each of the three different broadcasting regimes (direct, relay, transducted) expressed as sender and receiver nodal broadcasting strengths (WBS_sender_ and WBS_receiver_ respectively, both measured in bits/mm).

To further investigate the top regions involved in different broadcasting scenarios, we outlined the sender-receiver broadcasting changes (using WBS_sender_ and WBS_receiver_; see Methods section) for the top 10 brain regions in the three different communication regimes depicted in Figure 3. Overall, these regions corroborate the hypothesis of a regional specificity in the communication dynamics in large-scale human brain networks (Figure 4). Specifically, significant source-target asymmetries were found when brain regions were broadcasting in the transducted regime (Wilcoxon rank-sum test, *p* = 8.7 × 10^−05^ for the top 10 regions, *p* = 7.9 × 10^−16^ when testing across all 374 brain nodes); tendency toward asymmetry was found for the nodal broadcasting strength in the relay regime (Wilcoxon *p* = 1.8 × 10^−04^ for the top 10 regions, *p* = 0.67 when testing across all 374 brain nodes); finally, no significant source-target skewness in broadcasting was found in direct communication (Wilcoxon *p* = 0.66 for the top 10 regions, *p* = 0.91 when testing across all 374 brain nodes).

## DISCUSSION

Understanding how the brain processes information is one of the major challenges facing the neuroscientific community in the next decade. Nonetheless, the investigation advances across different temporal and spatial scales, from neuronal population (Quian Quiroga & Panzeri, 2009) to MRI-based connectomes (Avena-Koenigsberger et al., 2018; Marinazzo et al., 2014; Mišic´ et al., 2015). Information theory provides a well-established mathematic framework to explore the statistical dependencies present in brain data (Wibral, Lizier, & Priesemann, 2015; Wibral, Vicente, & Lizier, 2014).

What is still lacking, in our opinion, is a theory that would allow us to investigate the information carrying capacity of a *brain network.* MRI-based connectomes can indeed be modeled as a system of multiple dynamically interacting senders and receivers (Mišic´ et al., 2014a, 2014b, 2015; Tipnis et al., 2018). Exploration of the presence of different communication regimes in brain networks will introduce new elements and insights in brain communication problems, such as interference and cooperation and feedback between brain regions. Extending the communication problem to a brain network level can help our understanding of how communication dynamics relate to cognitive transitions and, ultimately, behavior.

In our investigation, we aim to contribute to the field by using information-theoretical tools for assessing communication dynamics in brain networks, based on their functional and structural topology. Here we introduced two information-theoretical measurements to account for communication transferred on top of a structural topology in human brain networks, specifically along the shortest paths connecting pairs of brain regions. Taking inspiration from Shannon’s seminal papers on communication, we defined PPS to serve as a quality index of how likely a shortest path is to take part in communication dynamics between a pair of regions. Using this score, we defined and explored three different regimes of communication in an MRI-based brain network: absent, relay, and transducted (Figure 2). Qualitative comparisons of communication regimes of resting-state and task (reasoning task) derived functional connectomes showed similar patterns emerging for the relay and transduction regimes, but not for absent paths (Figure 2B). This corroborates the idea of a relationship between communication dynamics and brain functional reconfigurations (Schultz & Cole, 2016). That is, depending on the “cognitive state” in which the brain operates, communication might diffuse along many diverse paths, not necessarily the shortest.

Additionally, we define a second measurement that is complementary to PPS, termed the path broadcasting strength (PBS), which is a measurement of the likelihood that communication along a path is being transferred or spread around to the neighboring nodes. Within each of the defined PPS regimes, with the addition of direct (single-edge) paths, we explored the broadcasting capacity of the resting-state connectome in the HCP dataset. Notably, we found subcortical regions (caudate, thalamus, and cingulum areas) to be *broadcaster hubs* in the direct communication regimes; the limbic system (amygdala and insula cortices) to be major *broadcast relay stations*; finally, the visual and ventral cortices to be primary centers of broadcasting transduction streams (Figure 3).

Inspired by a recent work (Seguin et al., 2019), we further explored this regional specificity by evaluating the asymmetry of broadcasting, for each communication regime, on the brain regions with highest nodal broadcasting strength. To do so, we distinguished those brain regions when being a target (receiver) or a source (sender). Interestingly, direct broadcasting showed greatest symmetry in paths originating/terminating primarily in subcortical nodes. Sender-receiver asymmetry becomes more pronounced in regions with a high broadcasting strength in relay, followed by transducted regime paths (Figure 4).

As a matter of fact, in the case of communication of directly connected nodes, top PBS regions were those of the subcortical and attention/default networks, and showed a similar PBS magnitude when serving as either a sender or a receive node. Striatal regions are known to receive direct inputs from brainstem and cortical regions, serving to integrate information related to motor function and reward (Haber, 2016). Attention related areas (retrosplenial cortex) have been demonstrated to be involved in learning and navigation, working in concert with thalamic and hippocampal regions (Vann et al., 2009), a function that is complementary to the striatal role in motor control. Therefore, based on the findings presented here, it is likely that the direct communication regime captures activity of nodes that receive several inputs, integrate the information, and send widespread outputs to higher order cortical regions with little augmentation of the signal (Choi et al., 2012).

For the relay transduction regime (i.e., paths where the signal is not or minimally transformed on its way from source to target), half of the top 10 regions belonged to the limbic network, 3 to subcortical, and 1 to each fronto-parietal and ventral attention network. These nodes were primarily in the temporal lobe (perirhinal ectorhinal, amygdala, piriform) and frontal lobe (inferior 68 transitional [approximately dorsolateral prefrontal] and area 25 [subcallosal]; Figure 4), with the remaining relay nodes belonging to left posterior insula and right pallidum. In this regime, PBS values are higher when they serve as the receiver in shortest paths, as compared to being a sender. This suggests that under the relay regime, arriving information has a greater specificity to the path traveled, compared to departing (sent out) information, which has greater tendency to spread out to neighboring nodes on the path. The default mode system is commonly thought of as being active at rest, or during passive tasks, where its temporal and frontal subsystems provide information for construction and flexible use of mental simulations, respectively (Buckner et al., 2008; Yeo et al., 2011). Interpretation of our results in the context of previous work on the DMN may hinge on the association between function and communication regime. In particular, a routing-like mode during retrieval of information from memory, and a broadcasting mode for construction and output of mental simulations (e.g., thinking about the future).

Transducted communication pathways, where signal undergoes modification on its path from source to target, showed greater broadcasting on paths where they were the source (as compared to target). Among the top regions in this regime were areas of the visual and default mode networks that were in some cases bilateral (Area V3A [visual]) or adjacent (left second and third visual areas [visual]; left area 31p ventral and area ventral 23a+b [limbic]; right fourth and eighth visual areas [visual]; Figure 4). Areas of the visual network receive highly specific visual input from their receptive visual field via the lateral geniculate nuclei of the thalamus. Upon reaching the visual cortex information is propagated out to other regions via processing streams that are involved in object recognition, motion, and representation in space, among others. In this regard, the information captured by PBS, from the joint structure/function connectomes, agrees with our neuroanatomical understanding of the visual system.

This study has some limitations. The impact of the brain parcellation on the definition of the communication regimes needs to be explored, as well as the choice of the soft boundaries between them (here defined on a resting-state DMN-based PPS distribution; see Methods for details); the effect of the uniform binning on the mutual information-derived connectomes should be further investigated, as well as the use of other information-based measurement of entropy between brain time series (e.g., transfer entropy or multivariate mutual information; Amico et al., 2017a; Schreiber, 2000). The use of resting state as a “null” condition or baseline for the tasks depends on several assumptions about neural activity during “rest” (Cole et al., 2014; Schultz & Cole, 2016). The effect of using different null conditions needs to be further investigated in terms of communication characteristics. Our study has focused on static functional connectomes as estimated by using the entire scanning length of each fMRI condition. Further studies should cover changes in communication and communication regimes within fMRI conditions by using the framework provided in this paper on dynamical functional connectivity (Hutchison et al., 2013; Preti et al., 2017; Shine et al., 2016). Finally, the choice of a reference baseline model to define the boundaries between the communication regimes (broken, relay, and transducted) is somewhat arbitrary. We introduced the use of DMN at rest as the functional subcircuit in which most of the communication should be identified as relay and hence establish relative associations of communication regimes with respect to the resulting PPS distribution (see Supporting Information Figure S1). However, other null models attending to a different rationale or also imposing different topological invariants might identify different PPS communication boundaries.

There are many possible extensions of this initial work on brain network information theory. For instance, the framework can be used on connectivity based on other modalities of brain data (as obtained via MEG, EEG, etc.) and can be extended to brain networks at different spatial scales (i.e., neuronal networks, mesoscopic brain networks). The utility of PPS and PBS for predicting behavioral, demographics, and/or clinical scores should also be further investigated. Finally, one might want to consider PPS and PBS along multiple paths or path ensembles, thus not restricting to the shortest ones (Avena-Koenigsberger et al., 2017), or even select the “best communication pathway” based on PPS (or a variation of it). In the context of this framework, concepts such as interference or cooperation and feedback (Mišic´ et al., 2015) may additionally be included in the model. Finally, while we used fiber length, structural contribution of streamline count or a combination of the two, might be considered as well.

In conclusion, we proposed a novel methodology, rooted in information theory, to investigate communication regimes and communication modes in large-scale brain networks (i.e., brain network information theory). This framework sets the ground for a better characterization of brain communication regimes and how they change as subjects perform different tasks.

## ACKNOWLEDGMENTS

Data were provided (in part) by the Human Connectome Project, WU-Minn Consortium (Principal Investigators: David Van Essen and Kamil Ugurbil; 1U54MH091657) funded by the 16 NIH Institutes and Centers that support the NIH Blueprint for Neuroscience Research, and by the McDonnell Center for Systems Neuroscience at Washington University. Joaquín Goñi acknowledges financial support Purdue Discovery Park Data Science Award “Fingerprints of the Human Brain: A Data Science Perspective.” Enrico Amico acknowledges financial support from the SNSF Ambizione project “Fingeprinting the Brain: Network Science to Extract Features of Cognition, Behavior and Dysfunction” (grant number: PZ00P2_185716).

## SUPPORTING INFORMATION

Supporting information for this article is available at https://doi.org/10.1162/netn_a_00185. The code used for computing PPS and PBS will be made available on the CONN*plexity* lab website (https://engineering.purdue.edu/ConnplexityLab).

## AUTHOR CONTRIBUTIONS

Enrico Amico: Conceptualization; Data curation; Formal analysis; Methodology; Writing – original draft; Writing – review & editing. Kausar Abbas: Methodology; Writing – original draft; Writing – review & editing. Duy Anh Duong-Tran: Formal analysis; Methodology; Writing – original draft. Uttara Tipnis: Methodology; Writing – original draft. Meenusree Rajapandian: Formal analysis; Writing – original draft. Evgeny Chumin: Formal analysis; Writing – original draft. Mario Ventresca: Conceptualization; Methodology; Writing – original draft. Jaroslaw Harezlak: Conceptualization; Funding acquisition; Writing – original draft. Joaquín Goñi: Conceptualization; Formal analysis; Funding acquisition; Investigation; Methodology; Supervision; Validation; Writing – original draft; Writing – review & editing.

## FUNDING INFORMATION

Joaquín Goñi, National Institutes of Health (https://dx.doi.org/10.13039/100000002), Award ID: R01EB022574. Jaroslaw Harezlak, National Institutes of Health (https://dx.doi.org/10.13039/100000002), Award ID: R01MH108467. Joaquín Goñi, National Institutes of Health (https://dx.doi.org/10.13039/100000002), Award ID: P60AA07611.

## TECHNICAL TERMS

Graph: An ordered pair formed by a set of nodes and a set of edges (which represent connections between pairs of nodes). Nodes are usually represented by circles, whereas edges are represented by lines or arcs connecting pairs of nodes.
Connectome or structural connectivity (SC) matrix: A network representation of the physical connections in the brain. Nodes represent brain regions, whereas edges represent physical connections of pairs of brain regions through the white matter. Weights typically denote the density or also the integrity of the connections.
Functional magnetic resonance imaging (fMRI): A noninvasive technique that estimates brain activity by detecting changes associated with blood flow. The rationale of this technique relies on the fact that there is a positive association between cerebral blood flow and neuronal activation.
Functional connectome/connectivity (FC) matrix: A network representation of the functional coupling between brain regions. Such coupling is usually measured by quantifying the statistical dependencies between time series of brain regions (e.g., pairwise Pearson’s correlation, mutual information) as obtained by functional magnetic resonance imaging (fMRI).
Search information: Measurement that quantifies the accessibility or hiddenness of the shortest path between a source node and a target node within the network by measuring the amount of knowledge or information (expressed in bits) needed to access that exact path.
Path processing score (PPS): Estimates how much a brain signal has changed or transformed on a specific path between a source and a target brain region. A negative score is indicative of a path that is not being used for communication; a PPS around zero indicates that information is passed almost intact along a path from the source to the target, whereas a high PPS indicates that the signal has gone through considerable transformation. Units are bits.
Path broadcasting strength (PBS): Estimates the propagation of the signal through the edges adjacent to a path being assessed (in this study we specifically assessed shortest paths). A low PBS indicates a routing-based communication along a path, whereas a high PBS indicates that the communication is not specific to that path, but is also being broadcast or propagated through neighboring edges. Units are bits/mm when using fiber length (mm) derived structural connectomes to establish the paths.
Diffusion-weighted imaging (DWI): A form of magnetic resonance imaging (MRI) technique based on measuring the random Brownian motion of water molecules within a voxel based on sampling 3D directions. This technique allows for estimating white matter streamlines and fiber bundles connecting brain regions.
Tractography: Computational reconstruction procedure that may be used to obtain, from DWI data, white matter streamlines or fiber tracts connecting different brain regions.
Nodal strength: In a weighted graph (i.e., where edges have assigned numerical values), it represents the total sum of the edge weights attached to a node.
Nodal broadcasting strength (WBS): The average of WBS_sender_ and WBS_receiver_. WBS_sender_ is the sum (strength) all PBS values when a brain region is a sender (source of a path). WBS_receiver_ is the sum (strength) all PBS values when a brain region is a receiver (target of a path).
