# Supplementary material for: Toward an information theoretical description of communication in brain networks
Source: Netw Neurosci. 2021 Aug 30;5(3):646–65. doi: 10.1162/netn_a_00185 (PMC8567835; doi:10.1162/netn_a_00185)
Supplement: Supplementary file 1 [file netn-05-646-s001.pdf]

Amico, E., Abbas, K., Duong-Tran, D. A., Tipnis, U., Rajapandian, M., Chumin, E., Ventresca, M., Harezlak, J., Goñi, J. (2021). Supporting information for "Towards an information theoretical description of communication in brain networks." *Network Neuroscience.* Advance publication. https://doi.org/10.1162/netn_a_00185

Supplementary Information

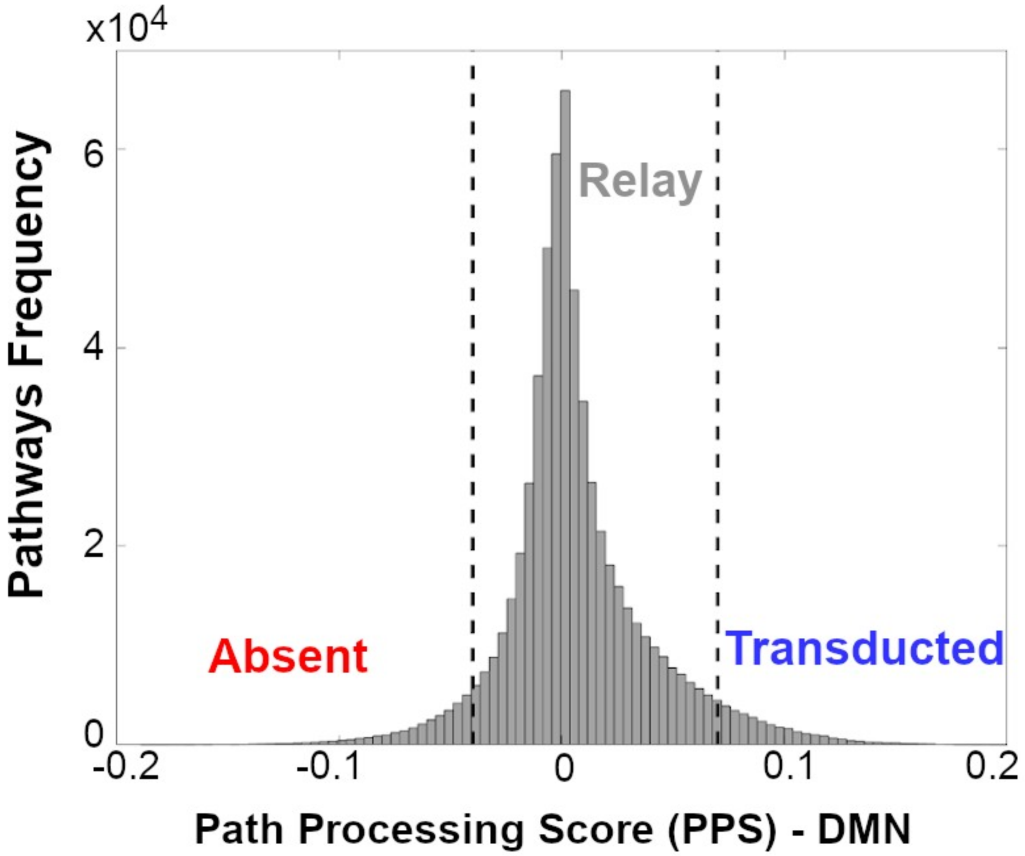

Figure S1. Relay baseline for path processing score (PPS) communication regimes. The DMN PPS distribution was obtained by considering the DMN-based shortest-paths and their corresponding (subject-level) PPS in resting state. Dashed vertical lines denote 5-95 percentiles respectively. Those percentiles were used as the range within which relay communications take place. Values below percentile 5 correspond to absent communication, whereas values above percentile 95 correspond to transducted communication.

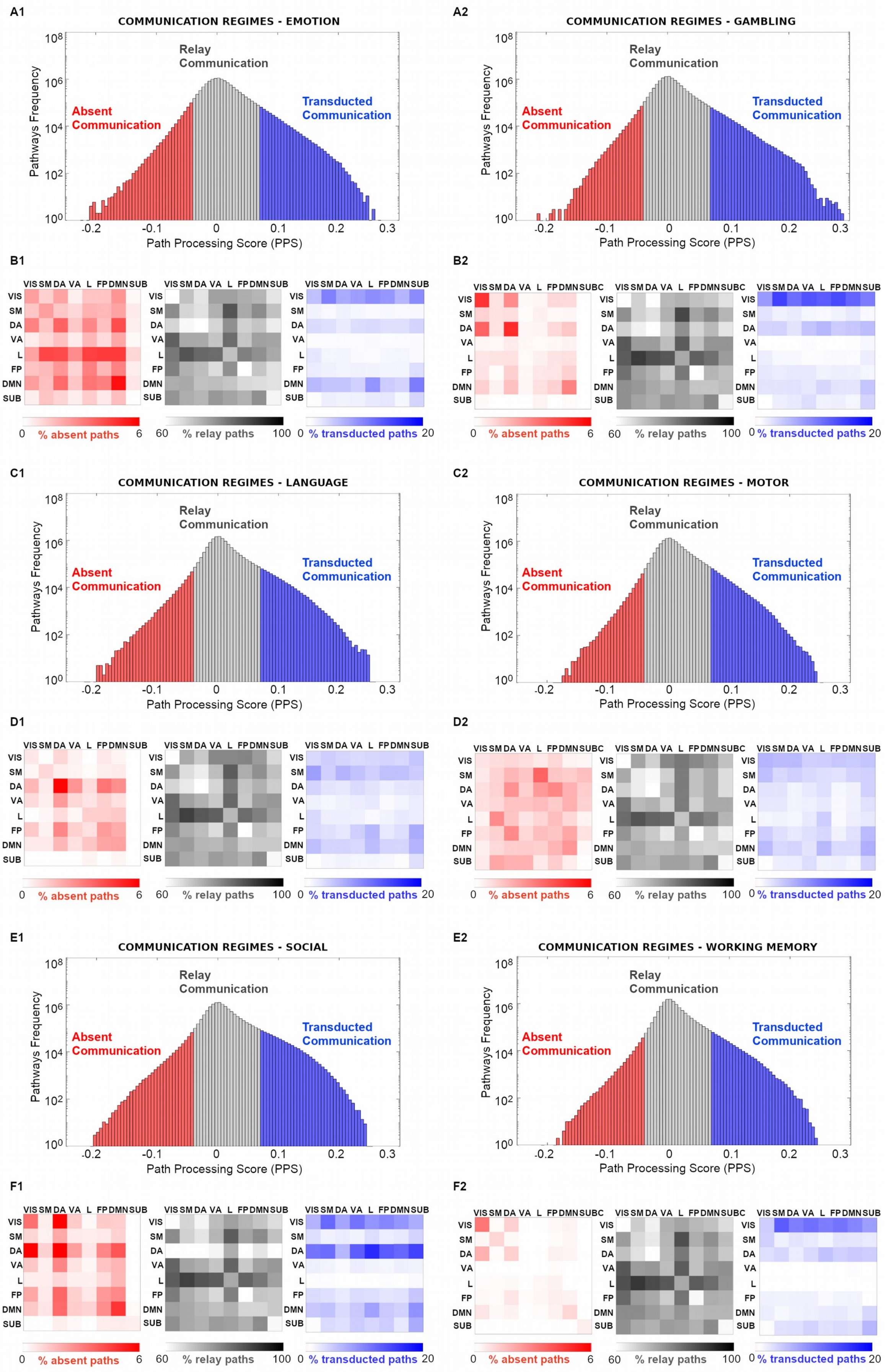
A1
COMMUNICATION REGIMES - EMOTION
Relay Communication
Absent Communication
Transducted Communication
Pathways Frequency
Path Processing Score (PPS)
A2
COMMUNICATION REGIMES - GAMBLING
B1
B2
VIS SM DA VA L FP DMN SUB
VIS SM DA VA L FP DMN SUBC
% absent paths
% relay paths
% transducted paths
C1
COMMUNICATION REGIMES - LANGUAGE
C2
COMMUNICATION REGIMES - MOTOR
D1
D2
E1
COMMUNICATION REGIMES - SOCIAL
E2
COMMUNICATION REGIMES - WORKING MEMORY
F1
F2

Figure S2. Communication regimes in large-scale brain networks. A1) Emotion task. Path Processing Score (PPS) on indirect pathways allows to separate brain network communication in three different regimes: absent, relay communication and transducted communication). **B1) Emotion task.** The percentage of paths, for the three different communication regimes, corresponding to the within and between 7 functional networks source-target pairs, as specified by (Yeo et al., 2011). An eighth sub-cortical community was added for completeness. Analogously, results are shown for **Gambling task (A2-B2)**, **Language task (C1-D1)**, **Motor task (C2-D2)**, **Social task (E1-F1)** and **Working Memory task (E2-F2).**

	MI bin-width 0.5	MI bin-width 0.75	MI bin-width 1	MI bin-width 2
MI bin-width 0.5	1	0.98	0.95	0.87
MI bin-width 0.75	0.98	1	0.98	0.90
MI bin-width 1	0.95	0.98	1	0.94
MI bin-width 2	0.87	0.90	0.93	1

Table S1. Effect of MI bin-width on MI-based functional connectomes. Table shows resting-state group average similarity between MI connectomes computed with different bin-widths (0.50, 0.75, 1 and 2, respectively). Note how the bin-width parameter minimally affects the MI connectome computation for the range of bin-widths explored.

	REST	***EMOT***	***GAMB***	***LANG***	***MOT***	***RELAT***	***SOC***	***WM***
***PPS* r**	0.98	0.96	0.96	0.95	0.93	0.97	0.97	0.97
PPS SD	0.017	0.021	0.019	0.017	0.018	0.021	0.019	0.016
PBS r	0.99	0.99	0.99	0.99	0.99	0.99	0.99	0.99
PBS SD	0.010	0.010	0.010	0.010	0.009	0.010	0.009	0.010

Table S2. Stability of PBS and PPS between runs and across subjects. Table reports the PPS and PBS group-average similarity across HCP trials (LR and RL, see Methods for details).
